# The Niagara Medical College

**Published:** 1885-02

**Authors:** 


### The Niagara Medical College.

This young and vigorous institution occupied the new building, erected for its special use, the first of January, and the lectures have been given, and dissections made, in its well-appointed rooms. The building is admirably adapted for its purposes, is conveniently arranged, and possesses all the facilities for the training and education of its students. We are happy to say that its classes are composed of excellent material and exhibit, in their progress, the careful and painstaking instruction which the faculty impart.

It is not too much to expect that the profession will soon learn to appreciate the efforts of this institution in the labor it bestows for the higher education of its students. The establishment of this college is now assured; its aims and purposes are for the benefit of the profession, and not of its faculty, and it is expected that the profession, already committed to improved systems of medical education, will support their efforts and liberally endorse the college in its mission of educating medical men thoroughly equipped for the duties and responsibilities of the profession.

---

We have before us the first number of the *Annals of Surgery*, edited by L. S. Pilcher, M. D., Brooklyn, and C. B. Keetley, F. R. C. S., of London, England, with an able corps of collaborators. As surgeons and as writers upon surgical subjects, both of the editors are well and favorably known. There is no other journal in the English language exclusively devoted to surgery. The contents of the initial number give promise that the *Annals of Surgery* will be a valuable addition to the library of every working surgeon. Subscription, $5.00 a year. Published by J. H. Chambers & Co., 405 North Third Street, St. Louis.

---

We have received, through the politeness of Prof. George E. Fell, the "Proceedings of the American Society of Microscopists," at its seventh annual meeting, held at Rochester, August, 1884. The volume contains 300 pages of matter, interesting especially to those who are devoted to microscopical investigations. It is highly creditable to our American scientists that their work enlists so much sympathy and co-operation at home and receives an appreciative recognition from the savants of the old world. This volume demonstrates that earnest work is directed in this important avenue of scientific inquiry.

